# Clear Lensectomy with Hydrus Stent in Black and Afro-Latino Patients: A 1-Year Retrospective Study

**DOI:** 10.1155/2022/8011745

**Published:** 2022-08-31

**Authors:** Daniel Laroche, Jessinta Oseni, Gideon Nkrumah, Chester Ng

**Affiliations:** ^1^Department of Ophthalmology, Icahn School of Medicine at Mount Sinai and New York Eye and Ear of Mount Sinai, New York, New York, USA; ^2^Advanced Eyecare of New York, New York, New York, USA; ^3^Albert Einstein College of Medicine, Bronx, New York, USA; ^4^University of Pittsburgh, Pittsburgh, Pennsylvania, USA

## Abstract

**Purpose:**

To determine the efficacy and safety of phacoemulsification, clear lensectomy, and the Hydrus microstent (Ivantis, Inc.) in Black and Afro-Latino glaucoma patients.

**Method:**

This is a retrospective nonrandomized study of 134 Black and Afro-Latino patients who underwent clear lensectomy with Hydrus stent implant for the treatment of glaucoma. For comprehensive analysis, patients were divided into mild, moderate, and advanced glaucoma. The evaluated parameters were reductions in the number of medications, intraocular pressure (IOP), mean deviation on visual field test, and visual acuity.

**Results:**

A total of 134 patients with 1-year follow-up were evaluated. At 1 year, the average number of medications significantly decreased from 2.5 ± 1.4 preoperatively to 0.43 ± 1.04 (*p* < 0.001) and IOP decreased from 14.4 ± 3.9 to 13.8 ± 3.10 (*p*=0.16). 110 (82.1%) patients were medication-free at 1 year (*n* = 57, 83.8% mild glaucoma; *n* = 37, 92.5% moderate glaucoma; *n* = 16, 61.5% advanced glaucoma). There was stabilization of mean deviation on the visual field test (baseline, −8.28; 1 year, −8.28; *p*=1). The most reported adverse effects were transient IOP spike and hyphema (*n* = 7, 5.2%; *n* = 3, 2.2%, respectively); both events were self-resolving. No decline in vision or sight-threatening complications were reported at 1 year, and no additional surgeries were required.

**Conclusion:**

This 1-year retrospective study demonstrated the efficacy and safety of clear lensectomy and Hydrus stent implantation in decreasing medication burden while maintaining lower IOP in Black and Afro-Latino glaucoma patients.

## 1. Introduction

Glaucoma is a leading cause of blindness worldwide [1]. Management of glaucoma often includes both medical and surgical interventions. The prevalence of glaucoma is higher in African and Afro-Latino patients compared with White patients [2]. Primary open-angle glaucoma (POAG) is the leading cause of irreversible blindness in African Americans; the disease also tends to be more rapidly progressive and with an earlier age of onset [1]. African Americans are less responsive to both medical and surgical treatment for POAG compared to other groups—this fact is exacerbated by socioeconomic inequalities that result in the limited accessibility of healthcare [1].

Trabeculectomy is vastly considered the gold standard for glaucoma surgery; however, this procedure is also associated with a higher risk of complications compared with current medical and laser therapy [2]. Over the past decade, cataract surgery and minimally invasive glaucoma surgery (MIGS) have evolved, showing improved efficacy, safety, and reduced rates of complications such as bleb-associated endophthalmitis, typically associated with traditional filtration surgeries [3]. The HORIZON study demonstrated a significant decrease in IOP with cataract surgery alone—from 25.3 ± 2.9 to 19.3 ± 4.2 mmHg. IOP reduction was even more pronounced in the group that underwent combined cataract surgery with Hydrus stent implantation—17.5 ± 3.9 mmHg [4].

We previously reported excellent outcomes with cataract extraction and MIGS in lowering IOP and medication burden in Black and Afro-Latino patients. In that study, the average age of the patients was 70.7 years, IOP decreased from 14.7 ± 3.7 mmHg to 13.9 ± 4.3 mmHg, and medication burden decreased from 2.6 ± 1.5 to 0.72 ± 1.4 [2]. We also reported short-term preservation of the visual field in this report. Recent data from the five-year HORIZON pivotal trial showed that the Hydrus microstent lowers the rate of visual field loss by 47% compared to cataract surgery alone [5].

The enlarging lens, secondary to aging or cataract, significantly impacts the pathophysiology of glaucoma [6]. Aging causes an increase in the lens' volume and width, which compresses the trabecular meshwork (TM) [7]. Furthermore, the increased lens volume also results in greater contact between the lens zonules and the posterior pigment epithelium; this contact is heightened during accommodation as there is posterior bowing of the iris and increased width of the lens. Increased iridolenticular contact leads to pigment liberation, further obstructing the trabecular meshwork [8]. After cataract extraction/lensectomy, Schlemm's canal expands and IOP decreases with the degree of expansion in adults [9]. Cataract surgery alone has been shown to significantly decrease IOP in patients with both acute closure (ACG) and primary open-angle glaucoma [7, 9–12].

This study reports our experience with earlier surgery, clear lensectomy, and the Hydrus stent in Black and Afro-Latino patients. The ethnic makeup of the patients is those that make up the local community in Harlem and Southeast Queens, New York City. The Eagle study showed the safety, efficacy, and cost-effectiveness of clear-lens extraction compared with laser peripheral iridotomy in patients with angle-closure glaucoma [13]. Our hypothesis is that clear lensectomy combined with Hydrus stent implantation would stabilize IOP and decrease patients' reliance on glaucoma medications. We have been performing clear lensectomy and Hydrus stent for both POAG and ACG, and here, we report our results.

## 2. Methods

This is a retrospective single-center study of 134 African-American and Afro-Latino glaucoma patients who underwent clear lensectomy in combination with the Hydrus microstent for the treatment of glaucoma. All patients were enrolled between December 2018 and October 2020 at a practice in New York, USA. The ethnicities of the study participants were representative of the neighboring community. Written informed consent for study participation was provided by all patients. The study complied with the tenets of the Declaration of Helsinki and was approved by the Institutional Review Board at the New York Eye and Ear Infirmary of Mount Sinai, New York, USA. HIPAA regulations were followed.

Patients had a comprehensive ophthalmological examination which included slit-lamp examination, Goldmann applanation tonometry, gonioscopy, visual acuity (VA), visual field (VF) examination with ZEISS Humphrey Visual Field Analyzer 3 (Dublin Inc.), and optic nerve assessment. The Hydrus stent implant is indicated for patients with medically treated glaucoma to reduce IOP and dependence on ocular hypotensive medications. This procedure avoids the risk and complications associated with traditional filtration surgery. Patients with open or moderately open angles were included in the study. The angles had to be open and/or be able to be opened with indentation gonioscopy or mild intermittent synechia of the angle to perform synechiolysis and insertion of the Hydrus stent after cataract surgery. The removal of the lens deepened the angle, creating more space for the stent. Patients with angle closure without visible TM, neovascular glaucoma, or uveitis were excluded from the study.

The primary outcome measures were IOP reduction and a decrease in dependency on ocular hypotensive medications. To determine the efficacy of clear lensectomy in combination with the Hydrus stent, patients were divided into subgroups which consisted of mild, moderate, and advanced glaucoma. These subgroups were created using the Hodapp–Parrish–Anderson criteria. Safety was assessed by evaluating reported adverse events from preoperative to final follow-up.

Patient information and surgical data were reviewed prior to the procedure. All IOP-lowering medications were discontinued in the operative eye on the day of procedure. Postoperatively, all patients were prescribed ofloxacin (Akorn) 0.3% one drop every 6 hours, ketorolac (Sandoz) 0.5% one drop every 8 hours, and prednisolone acetate (Sandoz) 1% ophthalmic solution one drop every 6 hours. Glaucoma medications were prescribed or withdrawn as deemed fit, and individual IOP reduction goals were designed for each patient. Clinical follow-up was assessed at day 1, 1 month, 3 months, 6 months, and 1 year.

### 2.1. Statistical Analysis

Data analysis was performed with Microsoft Excel for Mac (v. 16.16, Microsoft Corp). A paired *t*-test was used for analysis, and the significance level was set at *p* ≪ 0.05.

### 2.2. Medical Device

**Figure 1:**
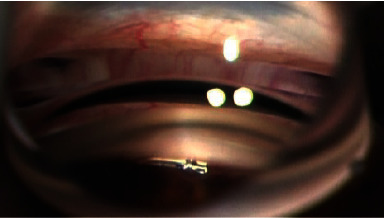
Hydrus stent inserted into Schlemm's canal.

The Hydrus stent is a device composed of biocompatible nickel-titanium alloy. It dilates and scaffolds a quadrant of Schlemm's canal and is modeled to fit the exact curvature of the canal. The device is inserted via the *ab interno* approach through the resistant TM into Schlemm's canal (Figure 1). With its orifice in the anterior chamber (AC), the Hydrus steadily drains aqueous fluid through the TM into the collecting channels via Schlemm's canal [14].

### 2.3. Procedure

**Figure 2:**
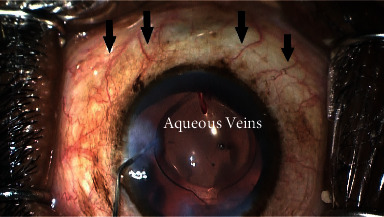
Aqueous veins visible before balanced saline solution placed through the Hydrus stent.

**Figure 3:**
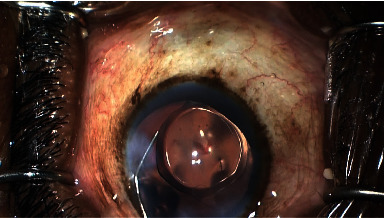
Blanching of aqueous veins with balanced saline solution through the Hydrus stent.

The operative eye was draped, and patients were administered topical anesthesia and betadine. A clear corneal temporal phacoemulsification was performed, and an intraocular lens was inserted. To maintain a deep angle, ophthalmic viscoelastic (EndoCoat, Abbott Inc.) was injected in the anterior chamber. Paracentesis was performed to the right of the corneal incision. The microscope was angled toward the surgeon at about 45 degrees, while the patient's head was positioned away from the surgeon at a 45-degree angle. A Gonio lens (Katena, Inc.) was used in combination with the microscope to get a direct view of the iridocorneal angle. In cases in which the view of the TM was partially obstructed by peripheral anterior synechiae, a cyclodialysis spatula was used to reveal the TM via a goniosynechialysis. To maintain visibility, viscoelastic was used as a heme tamponade. After TM incision, the Hydrus stent was inserted into Schlemm's canal (SC) and the delivery cannula was removed. To confirm outflow to aqueous veins, 1 cc of diluted moxifloxacin (Vigamox) and balanced saline solution (50/50) was injected through the paracentesis (Figures 2 and 3).

## 3. Results and Discussion

The average age for the 134 study participants was 67.9 ± 10.6 years (Table 1). The left eye-to-right eye procedure ratio was 1.16 : 1.0, and females outnumbered males in a 1.03 : 1.0 ratio. The average preoperative IOP was 14.4 ± 3.9 mmHg with an average use of 2.5 ± 1.4 IOP-lowering medications; 97% of patients were managed with one or more IOP-lowering medications, while 20.9% were treated with four or more. Postoperatively, there was a notable reduction in IOP at day 1, 3 months, and 6 months (*p* < 0.00010, *p*=0.0025, and *p*=0.0027, respectively; Table 2). Compared with preoperative values, there was substantial decline in the total amount of medications at each postoperative visit. At postoperative day 1, average IOP and number of medications were 11.9 ± 4.7 mmHg and zero medication, which is equivalent to a 17.4% decrease in IOP and 100% reduction in medication use, respectively. At 1-month follow-up, the IOP returned to baseline, while patients maintained a 94.8% reduction in the number of ocular hypotensive medications used. At 3 months, the IOP declined with an average IOP of 13.1 ± 2.9 (9.0% reduction from preoperative values) on 0.38 ± 0.94 (84.8% decrease from the baseline) medications. IOP remained steady at 6 months, with a mean IOP of 13.1 ± 2.8, *p*=0.0027, and an 83.2% reduction in the amount of ocular hypotensive medications (average medication at 6 months = 0.42 ± 1.03, *p* < 0.0001). At 1 year, patients had an overall decrease in IOP with a 4.2% drop from baseline (mean IOP at 1 year = 13.8 ± 3.1, *p*=0.16) while having a significantly decreased mean medication use of 0.43 ± 1.04 (82.8% drop from baseline); 82.1% of patients did not require any medication use.

Using the Hodapp–Parrish–Anderson criteria, patients were subdivided into mild, moderate, or advanced glaucoma, as presented in Table 3. Patients with mild glaucoma (*n* = 68) had a baseline average IOP of 15.0 ± 4.1 mmHg on 2.1 ± 1.18 medications. The average mean deviation on VF was −3.0 ± 1.7 dB. At 1-year follow-up, the mild glaucoma group experienced a 4.7% reduction in mean IOP and an 82% reduction in the number of medications used. Patients with moderate glaucoma (*n* = 40) had a preoperative mean IOP of 13.7 ± 3.9 mmHg on 2.6 ± 1.4 medications. The average mean deviation on VF was −8.6 ± 1.7 dB. The mean spherical equivalent was 0.18 ± 1.06 preoperatively and −0.44 ± 0.35. The mean IOP decreased by 2.9% in 1 year. The moderate glaucoma group experienced the most reduction in the number of medications—92.3% at 1 year. Patients with advanced glaucoma (*n* = 26) had a baseline mean IOP of 14.1 ± 3.6 mmHg on 3.2 ± 1.5 medications and an average mean deviation on VF of −20.7 ± 5.3 dB. Postoperatively, the average IOP reduced by 2.8% in 1 year. The advanced glaucoma group experienced a 72.5% medication reduction at 1 year. The percent of patients on no medications increased from 3% at baseline to 82.1% at 1 year (mild, 83.8%; moderate, 92.5%; and advanced, 61.5%).

A separate analysis of POAG and ACG patients was performed (Table 4). The ACG group experienced a statistically significant reduction in IOP (*p*=0.04) at 1 year compared to the POAG group. Both groups experienced a statistically significant reduction in medication use. The percent of patients on no medications increased from 2.9% at baseline to 84.5% at 1 year in the POAG group and from 0% at baseline to 70.8% at 1 year in the ACG group.

In all cases, the angle was already opened nasally after lensectomy or mechanically opened breaking mild intermittent peripheral anterior synechia using synechiolysis with a cyclodialysis spatula for Hydrus implantation. Postoperatively, some IOP spikes (IOP increase greater than 10 mmHg or IOP ⩾ 30 mmHg) and hyphema were noted and addressed. All adverse events were self-limited and nonvision threatening and required no secondary interventions. Usually within the first postoperative week, the blood that refluxed into the anterior chamber after Hydrus stent insertion spontaneously resolved.

### 3.1. Discussion

This retrospective noncomparative study of 134 Black and Afro-Latino patients demonstrates the efficacy of clear lensectomy in combination with Hydrus stent implantation for reducing IOP and decreasing medication dependence. All patients had an overall decrease in IOP of 4.2%, with 82.8% of patients medication-free at 1 year. Each subgroup also demonstrated a decreasing reliance on medications while maintaining lower IOPs. The moderate glaucoma group had the highest reduction in the number of medications, with a 92.3% drop from baseline and 92.5% of patients medication-free at 1 year. Fea et al. was able to show similar results in the multicenter study of open-angle glaucoma patients who underwent phacoemulsification with Hydrus stent placement [15]. Patients had a mean IOP of 15.7 ± 2.5 mmHg (19% drop from baseline), and 64% of patients were medication-free at 2 years' follow-up.

In the HORIZON study, the largest randomized controlled trial for an MIGS device with cataract surgery, the mean IOP was 16.7 ± 3.1 mmHg in the Hydrus group and 17.0 ± 3.4 mmHg in the control group and the number of glaucoma medications was 0.4 ± 0.8 in the Hydrus group and 0.8 ± 1.0 in the control group (*p* < 0.001) at 3 years' follow-up [16]. 73% of the Hydrus group were medication-free compared with 48% in the control group (*p* < 0.001). More recently, the 5-year follow-up results showed that 73% of “mild” Hydrus stent patients (one glaucoma medication at baseline) remained medication-free at five years compared with 48% of patients in the cataract surgery-alone group; the Hydrus group also experienced a 20–30% improvement in the medication-free rate compared with the control group [5]. The rate of subsequent invasive glaucoma surgery was 2.5% in the Hydrus group and 6.4% in the cataract surgery-alone group, which is more than a 60% reduction in the likelihood of requiring additional invasive glaucoma surgery. There was no significant increase in endothelial cell loss which remained stable in the Hydrus group compared to the control group [5].

Our study demonstrates the efficacy and safety of clear lensectomy and Hydrus stent implantation in Black and Afro-Latino patients with mild, moderate, and advanced glaucoma. As aging causes an increase in the lens volume which compresses the trabecular meshwork [6], clear lensectomy with the Hydrus microstent provides earlier therapeutic intervention and prevents disease progression. Our patients had an average age of 67.9 years, which is less than the typical age for cataract surgery (73 years). Patients with mild glaucoma experienced the greatest effect in IOP reduction at 1 year compared with patients with advanced glaucoma. All patients were able to maintain lower IOPs on either reduced or no pharmacologic agents; more than 60% of patients in each group were medication-free at 1-year follow-up, and no additional surgeries were required. In general, patients reported improved clarity in vision with better intermediate vision. Of the two patients that had decreased vision, one had advanced glaucoma with 20/40 preoperative vision and had an IOP spike with corneal edema during postop visits and recent refraction yielded 20/50. The second patient had significant optic disc pallor and was referred to a neuro-ophthalmologist to rule out a neuro-ophthalmic cause of decreased central vision.

Reported complications of the Hydrus stent placement include device malposition, device obstruction, transient IOP spike transient hyphema, peripheral anterior synechiae, and secondary surgical interventions [14, 17, 18]. In this study, the only reported complications of the Hydrus microstent were transient IOP spike (7 patients) and hyphema (3 patients). There were no complications for cataract surgery such as cystoid macular edema, endophthalmitis, or other related complications in this group.

Early control of IOP is important in slowing glaucoma progression and preserving vision. Black and Afro-Latino patients have a higher incidence of glaucoma and are also at a greater risk of developing blindness from the disease [19]. Due to limited healthcare accessibility and increasing medication costs which contribute to lack of medication adherence among these patients, we believe earlier surgical intervention with a nuanced therapeutic approach when needed is imperative in the management of glaucoma patients over 50 years.

In the United States, health insurance companies typically do not approve cataract surgery in patients with better than 20/40 vision. However, considering the efficacy and safety of this procedure, these companies should authorize earlier cataract surgery/clear lensectomy combined with MIGS in glaucoma patients over 50 years as this procedure will ensure a decrease in medication burden and disease progression by removing the offending agent of the enlarged lens and restoring outflow by bypassing the obstructed trabecular meshwork and restoring outflow to Schlemm's canal.

## 4. Conclusion

Clear lensectomy surgery with the Hydrus microstent implantation resulted in a significant decrease in the use of ocular hypotensive medications in Black and Afro-Latino patients with glaucoma. This procedure also demonstrated an exceptional safety profile as adverse effects were minimal and self-limiting. Study limitations include its retrospective study design, lack of control group of stand-alone phacoemulsification or Hydrus, small sample size, and limited follow-up. However, the study demonstrates the efficacy of clear lensectomy in combination with the Hydrus microstent in Black and Afro-Latino glaucoma patients. Future research with larger sample sizes and longer follow-up is required to corroborate the efficacy and safety illustrated in our study.

## Figures and Tables

**Table 1:** Baseline characteristics of patients who underwent clear lensectomy in combination with Hydrus microstent implant.

Variable	Category	Statistics
Age (years)	Mean (SD)	67.9 ±10.6
Sex, *n* (%)	Male	66 (49.3%)
	Female	68 (50.7%)
Eye, *n* (%)	Right	62 (46.3%)
	Left	72 (53.7%)
Baseline IOP (mmHg)	Mean (SD)	14.4 ± 3.9
Ocular hypotensive medications	Mean (SD)	2.5 ± 1.4
Patients using ocular hypotensive medications, *n* (%)	0	4 (3.0%)
	1	39 (29.1%)
	2	25 (18.7%)
	3	38 (28.4%)
	≥4	28 (20.9%)
Visual acuity (logMAR)	Mean ± SD	0.12 ± 0.04
MD on VFT (dB)	Mean ± SD	−8.28 ± 7.26
Type of glaucoma	POAG	103 (76.9%)
	ACG	24 (17.9%)
	Pigmentary	1 (0.7%)
	Mixed	6 (4.5%)

ACG, angle-closure glaucoma; IOP, intraocular pressure; MD, mean deviation; *n*, number of patients; POAG, primary open-angle glaucoma; SD, standard deviation; VFT, visual field test.

**Table 2:** Primary outcome measures—IOP, CDVA, number of medications, and mean deviation on visual field at preoperative and follow-up visits for all patients.

Timepoint	IOP (mmHg)			Ocular hypotensive medications			CDVA	Visual field test	
	*N*	Mean ± SD	*p* value	*N*	Mean ± SD	*p* value	LogMAR (mean ± SD)	*p* value	Mean deviation ± SD (dB)
Preoperative	134	14.4 ± 3.9	—	134	2.5 ± 1.4	—	0.12 ± 0.04	—	−8.28 ± 7.26
Postoperative day 1	132	11.9 ± 4.7	<0.00010^*∗*^	—	—	—	—	—	—
1 month	132	14.6 ± 4.90	0.71	132	0.13 ± 0.63	<0.00010^*∗*^	0.11 ± 0.2	<0.00010^*∗*^	—
3 months	114	13.1 ± 2.90	0.0025^*∗*^	115	0.38 ± 0.94	<0.00010^*∗*^	0.08 ± 0.19	<0.00010^*∗*^	—
6 months	111	13.1 ± 2.80	0.0027^*∗*^	111	0.42 ± 1.03	<0.00010^*∗*^	0.09 ± 0.2	<0.00010^*∗*^	—
1 year	134	13.8 ± 3.10	0.16	134	0.43 ± 1.04	<0.00010^*∗*^	0.09 ± 0.2	<0.00010^*∗*^	−8.28 ± 7.70 (*p* > 0.99> 0.99)

CDVA, corrected distance visual acuity; IOP, intraocular pressure; *N*, number of patients; SD, standard deviation. ^*∗*^*p* values are statistically significant.

**Table 3:** Intraocular pressure and number of medications at preoperative and follow-up visits for patients with mild, moderate, and advanced glaucoma who underwent clear lensectomy in combination with Hydrus microstent implant.

Timepoint	Mild glaucoma (*N* = 68)				Moderate glaucoma (*N* = 40)				Advanced glaucoma (*N* = 26)			
	IOP (mean ± SD; mmHg)	*p* value	Number of medications (mean ± SD)	*p* value	IOP (mean ± SD; mmHg)	*p* value	Number of medications (mean ± SD)	*p* value	IOP (mean ± SD; mmHg)	*p* value	Number of medications (mean ± SD)	*p* value
Preoperative	15 ± 4.1	—	2.1 ± 1.18	—	13.7 ± 3.9	—	2.6 ± 1.4	—	14.1 ± 3.6	—	3.2 ± 1.5	—
Postoperative day 1	11.9 ± 4.9	<0.001^*∗*^	—	—	11.5 ± 4.3	0.02^*∗*^	—	—	12.6 ± 5.1	0.22	—	—
1 month	15 ± 5.5	>0.99	0.11 ± 0.61	<0.001^*∗*^	14.2 ± 4.7	0.60	0.03 ± 0.16	<0.001^*∗*^	14.3 ± 3.4	0.83	0.3 ± 1.0	<0.001^*∗*^
3 months	13.5 ± 3.0	0.016^*∗*^	0.28 ± 0.88	<0.001^*∗*^	12.5 ± 2.6	0.11	0.35 ± 0.91	<0.001^*∗*^	13.1 ± 2.8	0.26	0.71 ± 1.1	<0.001^*∗*^
6 months	13.6 ± 3.1	0.02^*∗*^	0.41 ± 1.12	<0.001^*∗*^	12.4 ± 2.3	0.08	0.24 ± 0.8	<0.001^*∗*^	12.9 ± 2.8	0.18	0.72 ± 1.1	<0.001^*∗*^
1 year	14.3 ± 3.1	0.26	0.40 ± 1.05	<0.001^*∗*^	13.3 ± 2.4	0.58	0.2 ± 0.72	<0.001^*∗*^	13.7 ± 3.8	0.69	0.88 ± 1.3	<0.001^*∗*^

IOP, intraocular pressure; *N*, number of patients; SD, standard deviation. ^*∗*^*p* values are statistically significant.

**Table 4:** Intraocular pressure and number of medications at preoperative and follow-up visits for patients with primary open-angle and angle-closure glaucoma who underwent clear lensectomy in combination with Hydrus microstent implant.

Timepoint	POAG (*N* = 103)					ACG (*N* = 24)				
	IOP (mean ± SD; mmHg)	*p* value	Number of medications (mean ± SD)	*p* value	Mean deviation ± SD (dB)	IOP (mean ± SD; mmHg)	*p* value	Number of medications (mean ± SD)	*p* value	Mean deviation ± SD (dB)
Preoperative	14 ± 3.5	—	2.5 ± 1.4	—	−9.02 ± 7.56	15.75 ± 5.2	—	2.62 ± 1.20	—	−6.65 ± 5.53
Postoperative day 1	12.1 ± 4.9	<0.001^*∗*^	—		—	10.4 ± 2.5	<0.001^*∗*^	—		—
1 month	14.7 ± 4.7	0.22	0.16 ± 0.72	<0.001^*∗*^		14.5 ± 6.1	0.44	0	<0.001^*∗*^	
3 months	12.8 ± 2.6	0.007^*∗*^	0.37 ± 0.94	<0.001^*∗*^		13.6 ± 3.7	0.12	0.57 ± 1.0	<0.001^*∗*^	
6 months	13.0 ± 2.7	0.02^*∗*^	0.33 ± 0.89	<0.001^*∗*^		13.3 ± 3.5	0.06	0.86 ± 1.4	<0.001^*∗*^	
1 year	13.8 ± 3.06	0.66	0.34 ± 0.92	<0.001^*∗*^	−8.69 ± 7.80 (*p*=0.76)	13.2 ± 2.9	0.04^*∗*^	0.91 ± 1.5	<0.001^*∗*^	−7.88 ± 7.58 (P = 0.53)

ACG, angle-closure glaucoma; IOP, intraocular pressure; *N*, number of patients; POAG, primary open-angle glaucoma; SD, standard deviation. ^*∗*^*p* values are statistically significant.

## Data Availability

The datasets analyzed during the study are available from the corresponding author on reasonable request.
